# Alternative Experimental Models for Studying Influenza Proteins, Host–Virus Interactions and Anti-Influenza Drugs

**DOI:** 10.3390/ph12040147

**Published:** 2019-09-30

**Authors:** Sonja C. J. H. Chua, Hui Qing Tan, David Engelberg, Lina H. K. Lim

**Affiliations:** 1 Department of Physiology, Yong Loo Lin School of Medicine, National University of Singapore, Singapore 117593, Singapore; 2 NUS Immunology Program, Life Sciences Institute, National University of Singapore, Singapore 117456, Singapore; 3 CREATE-NUS-HUJ Molecular Mechanisms of Inflammatory Diseases Programme, National University of Singapore, Singapore 138602, Singapore; 4 Department of Microbiology, Yong Loo Lin School of Medicine, National University of Singapore, Singapore 117545, Singapore; 5 Department of Biological Chemistry, The Institute of Life Science, The Hebrew University of Jerusalem, Jerusalem 9190401, Israel

**Keywords:** influenza, MDCK, A549, ferrets, mice, pigs, macaque, yeast, *S. cerevisiae*, *Drosophila*, zebrafish, human nasal epithelial cells, human bronchial epithelial cells, alveolar epithelial cells

## Abstract

Ninety years after the discovery of the virus causing the influenza disease, this malady remains one of the biggest public health threats to mankind. Currently available drugs and vaccines only partially reduce deaths and hospitalizations. Some of the reasons for this disturbing situation stem from the sophistication of the viral machinery, but another reason is the lack of a complete understanding of the molecular and physiological basis of viral infections and host–pathogen interactions. Even the functions of the influenza proteins, their mechanisms of action and interaction with host proteins have not been fully revealed. These questions have traditionally been studied in mammalian animal models, mainly ferrets and mice (as well as pigs and non-human primates) and in cell lines. Although obviously relevant as models to humans, these experimental systems are very complex and are not conveniently accessible to various genetic, molecular and biochemical approaches. The fact that influenza remains an unsolved problem, in combination with the limitations of the conventional experimental models, motivated increasing attempts to use the power of other models, such as low eukaryotes, including invertebrate, and primary cell cultures. In this review, we summarized the efforts to study influenza in yeast, *Drosophila*, zebrafish and primary human tissue cultures and the major contributions these studies have made toward a better understanding of the disease. We feel that these models are still under-utilized and we highlight the unique potential each model has for better comprehending virus–host interactions and viral protein function.

## 1. Introduction

### 1.1. Ninety Years after Its Identification, the Influenza Virus Has Not Been Eradicated

Almost ninety years after the virus causing the disastrous influenza epidemics was identified [1], 3–5 million people are still affected annually with severe symptoms and 500,000 die [2]. Furthermore, highly virulent strains still appear and result in local epidemics or global pandemics, such as the 2005 H5N1 Bird flu and the 2009 H1N1 Swine flu [3]. This situation is frustrating because at the same time, mankind has been more successful at tackling other viral maladies such as smallpox, measles and polio [4], and developed efficient drugs and vaccines against other disease-causing and pandemic-causing agents [5].

Indeed, significant advances have been made in understanding influenza. Major mechanistic aspects of the viral infection process and of the viral life cycle have been revealed [6] and important progress has been made in vaccination and therapy [7,8]. However, the fact that influenza still prevails demonstrates, unambiguously and painfully, that these drugs and vaccines are not as efficient as the vaccination and therapies of other diseases. Multiple reasons for this inefficiency in vaccination and therapy exist. Influenza viruses have high mutation rates and this seems to be a primary hurdle for the development of efficient and effective vaccines. These mutations could also account for the rapid evolution of drug-resistant influenza strainsb (C8)(C9) [6]. Given that influenza is a RNA virus, its RNA polymerase lacks proofreading activity and is more error-prone [9]. Also, as influenza is able to infect a variety of hosts, this increases the chance of mutation and genetic changes in the virus [6]. 

Another reason could simply stem from the fact that we still do not fully understand critical steps in the infection cycle and have not thoroughly unraveled the function and mechanisms of action of several of the influenza viral proteins. These latter aspects are being approached in a variety of experimental systems, primarily cell lines and mammalian and avian animal models. 

The fact that after 90 years of massive efforts, there is still no efficient therapy or comprehensive vaccination, combined with the many questions regarding influenza pathology that are still open, has motivated investigators to try new, in some cases seemingly irrelevant, experimental models. Indeed, models such as single-cell organisms (yeast), or invertebrates, such as *Drosophila melanogaster* (*Drosophila*) and aquatic vertebrates, such as zebrafish, appear to be not legitimate for studying a human-infecting virus. They do provide, nevertheless, advantages, which have contributed to our understanding of important aspects of viral-host interactions and viral replication [10,11]. Some of these advantages are unique to these systems and are derived from the fact that they are lower organisms in the evolutionary tree and consequently much simpler relative to mammals. An advantage that is derived from this simplicity is the widespread use of these organisms in basic biological research, which has led to the generation of sophisticated technology, libraries, mutants and information about the system, which are readily available. Some of these systems are also permissive for efficient and rapid genetic screens and for drug screening. The need for genetic screens and other large-scale approaches is essential to identify the numerous effects of the virus on host systems, achieved through close functional and structural connections with the biochemical machinery of the host. In addition to the simple animal models, another less used system in influenza research is that of primary human tissue cultures [12]. The relevance of such cells is obvious and they may replace some of the many studies that use cell lines of dogs and mice, and the human cell lines derived from cancerous cells. A further advantage of using primary cultures is that they can be isolated from patients and can be used for studying the disease at an individual level.

The purpose of this review is to draw attention to the emerging ‘alternative models’ and discuss the benefits one could extract from them for better comprehending the intricacies of the influenza virus and developing tools for devising novel anti-influenza therapies. 

### 1.2. The Life Cycle of the Influenza Virus

The Influenza virus belongs the *Orthomyxoviridae* family, which are negative-sense single-stranded RNA viruses that cause respiratory diseases [13]. There are three types of influenza viruses, named A, B and C. The most prolific member is Influenza A, which infects a large number of hosts and is the main cause of the seasonal and pandemic influenza [3]. Its genome is composed of symmetrical helixes in eight segments that are numbered according to their decreasing lengths. The segments encode surface glycoproteins [haemagglutinin (HA) and neuraminidase (NA)], matrix protein (M1), matrix ion channel (M2), viral RNA-dependent RNA polymerase complex [composed of nucleoprotein (NP), polymerase basic subunits (PB1 and PB2) and polymerase acid subunit (PA)], and nuclear export protein (NEP/non-structural protein NS2) [14]. Segments of some influenza A virus strains may encode a second or third polypeptide in alternative reading frames [15]. These proteins, such as PB1-F2 and PA-X, are functional and are known to be immunomodulatory, modulating the host response to the virus [16]. The influenza A virus can be further classified into different subtypes based on their HA and NA glycoproteins. Of the 18 haemagglutinin and 11 neuraminidase subtypes known today [17], only the H1, H2, H3, N1 and N2 subtypes have caused epidemics in humans [18]. 

The influenza virus life cycle can be divided into the following stages: host cell entry, uncoating, viral ribonucleoproteins (vRNPs) entry into the nucleus, transcription and translation of the viral genome, vRNPs export from the nucleus, viral assembly at the host cell and budding through the plasma membrane [19]. Viral HA binds to sialic acids on the cell surface membrane and this results in virus internalization via an endosome. The acidification within the endosome (assisted by the M2 ion channel) induces the viral envelope to fuse with the endosome. The fusion releases viral contents, which are the genetic material in the form of vRNPs. Viral replication occurs via synthesis of the positive-sense intermediate. Transcription of negative RNA into positive sense mRNA creates the proper platform for translation of viral proteins. Viral proteins and vRNPs migrate to the plasma membrane and are bundled into new virions. The new virions then bud from the plasma membrane to infect other cells [6]. 

### 1.3. The Disease of Influenza and Available Therapies

The Influenza virus is transmitted by respiratory droplets and causes acute respiratory diseases in humans. Young children, the elderly, those with chronic conditions like diabetes, heart disease and kidney disease, and immunocompromised patients are especially vulnerable to severe influenza [2,20]. Severe influenza may result in complications such as acute bronchitis and secondary bacterial pneumonia [21]. Influenza viruses evolve constantly with antigenic shifts (reassortment of viral segments, resulting in dramatically different viruses) and drifts (small antigenic changes to increase immune evasion). Due to viral adaptation and reassortment, highly virulent strains may appear and result in local epidemics or global pandemics such as the 1918 H1N1 Spanish pandemic, 2005 H5N1 Bird flu and the 2009 H1N1 Swine flu [3]. 

To date, only two antiviral drug classes, with some usefulness, are available and very few are under development or in trials [8]. The drugs available in the clinic are Matrix 2 (M2) ion channel inhibitors, (e.g., amantadine and rimantadine) and neuraminidase (NA) inhibitors (e.g., oseltamivir and zanamivir) [6]. The emergence of resistant virus strains have rendered M2 inhibitors useless and there is also growing resistance against NA inhibitors such as the H274Y mutation found in the 2009 H1N1 strain that made the virus oseltamivir-resistant [3,22]. However, there is an obvious fitness cost to viruses holding NA resistance which results in low frequency of such mutations over the years. Newer drugs, such as favipiravir, a nucleotide analog that targets the viral polymerase approved in Japan, and baloxavir marboxil, which is a cap-dependent endonuclease inhibitor, have been approved in Japan and the US [23], with pimodivir, a PB2 inhibitor, having pre-approval access by the FDA (NCT03834376). Some new drugs are being tested (C10)(C11) in clinical trials, including DAS181 (removes sialic acid receptors from glycan structures), Nitazoxamide (interferes with HA assembly) and HA monoclonal antibodies, such as VIS410 and MHAA4549A [24]. There are potential drugs, such as a class of compounds called naphthalimides, which antagonize NS1. This class of compounds has yet to undergo future development and reach clinical trials [25]. 

Given the scarcity of efficient anti-influenza drugs, the major strategy to prevent or control seasonal influenza epidemics is the annual influenza vaccination programme. Due to its varied effectiveness year to year, pandemics and epidemics still appear when the vaccine is mismatched with the predominant antigenic strains. One of the main methods to produce the influenza vaccine is through fertilized embryonated chicken eggs. First, the circulating virus is reassorted with a high-growth strain (A/Puerto Rico/8/1934). The vaccine strain is selected for high growth properties and contains HA and NA derived from the circulating strain. The selected strain is then amplified in eggs and the virus is harvested and inactivated. A serious disadvantage of the method is that the virus can become mismatched due to viral adaptation to egg culture, which affects vaccine efficacy [6]. Another upcoming method of vaccine production is using cell culture to propagate the virus. However, so far to date, only one vaccine, Flucelvax, has been approved by the FDA. Also, given that the vaccine was produced from egg-derived virus strains, egg adaptations may still exist in this vaccine [26]. Notably, the new experimental models described in this review could assist not only in understanding the virus biochemistry and physiology, but also in overcoming the current limitations of vaccine production. For example, this can be done via surface display in yeast [27] and the production of large quantities of viral antigens in yeast and *Drosophila* cells rather than propagating the virus in egg culture [28].

## 2. Traditional Models to Study Influenza

### 2.1. No Current Laboratory Model Has Fully Reproduced All Aspects of Human Viral Infection

The first animals that investigators were able to infect with the human influenza virus were ferrets [1] and this model remains in until today [29]. Yet, as convenient experimental models were required, rodents, including mice, hamsters, cotton rats and guinea pigs, became widely used [30]. Pigs are also used because the pathology of influenza in their lungs is similar to its manifestation in humans [31]. Pigs are even suspected as intermediate hosts of the virus [32]. Models that are evolutionarily closer to human, and therefore considered more relevant, are nonhuman primates, such as various macaques (Rhesus, Pig-tailed and Cynomolgus), Squirrel monkeys and African green monkeys (reviewed in Bouvier et al., 2015 [29] and Thangavel et al., 2014 [33]). In addition, avian models, such as chickens, are also used, but mainly for veterinary purposes [34].

The use of mammalian and avian models seems to be a logical first choice, under the assumption that the influenza virus naturally infects these models. It is also further assumed that the infection machinery and the life cycle of the virus within these model organisms are similar to that in humans. Notably, however, these assumptions do not hold for all models. For example, while ferrets are highly susceptible to the virus and are a good model from this perspective [35], high cost and lack of widely available resources such as antibodies limit its use. For mice, influenza virus could cause disease in mice if the virus is first adapted to this species by serial passages in the lung [30]. Thus, although mice are widely used as a model organism for influenza, primarily because it is cost-effective and permissive for genetic manipulation and has many mutant and transgenic animals available, mice might not be the ideal model for studying influenza due to differences in disease pathogenesis and limited infective viral strains [30]. Also, in some monkeys, while the virus does replicate in pulmonary cells, seasonal strains do not cause pathogenic symptoms (unless highly virulent viral strains are used) [30]. Thus, as even nonhuman primates cannot fully reproduce human viral infection, there is no optimal animal model system that manifests all aspects of the human disease. 

In the course of the establishment of immortal cell lines, some of those have been found to be infectable with influenza. Prominent examples are Madin-Darby Canine Kidney (MDCK), Vero cells, Human Embryonic Kidney (HEK) 293 and lung epithelial cells such as A549 and Calu-3. In addition to the models used for studying the entire scope of infection and viral life cycle, new insights into influenza could be derived from studying the intrinsic biochemical properties, structure-function relationships and specific functions of each viral protein. These aspects cannot be studied in the context of cellular or animal infection as the proteins may be regulated and controlled by the complex mammalian machinery. 

Therefore, to study the properties and functions of viral proteins, either the intrinsic features or those that appear following interaction with other proteins (viral or human), isolated systems, including in vitro systems have been applied. Influenza proteins have been expressed and purified as recombinant proteins from different sources, including bacteria and mammalian cells. Most proteins were assayed in vitro and some were crystalized [36,37,38,39,40]. These experimental systems are not unique to viral proteins, but are rather common techniques in biological research and will therefore not be described here. Yet, the study of the biochemistry of each viral protein could be advanced through the use of the alternative models discussed in the review. 

Below, we describe in brief the current models and elaborate on new models. 

### 2.2. Cell Lines

Influenza was initially shown to infect human embryo lung and kidney and primary monkey kidney cells [41,42], but it was later shown to be capable of infecting and replicating in various established cell lines of different organisms (reviewed in Green I.J. et al., 1957 [42]). Also, cell lines that cannot be infected could still absorb the virus upon transfection of viral DNA using reverse genetic approaches where plasmids containing viral cDNA are introduced into the cells [43]. Some cell lines are far more widely used than others, mainly due to better replication of the virus or because of their physiological relevance. In addition, there are current efforts to use various cell lines such as MDCK and HEK293 cells for vaccine production (reviewed in Milian E., et al., 2015 [44]). The most widely used, MDCK and the immortal lung-derived cell lines A549 and Calu-3, HEK293 and Vero cells, are described below.

#### 2.2.1. Madin-Darby Canine Kidney (MDCK) Cells

The Madin-Darby Canine Kidney (MDCK) cell line was established in 1958 by Madin and Darby [45], and was shown soon afterward to be susceptible to infection by various strains of influenza [46,47]. Apparently, other cell lines, such as kidney cells derived from bovine, chicken and monkeys and even human, were not permissive to viral infections or did not support many cycles of viral replications [42]. 

Based on the observation that MDCK cells support multiple rounds of virus replication, up to 35 serial passages, it was proposed that dogs might be natural hosts of the virus [46]. Since then, several MDCK derivatives have been developed to study influenza in vitro [45,48,49,50,51], primarily as an alternative method to embryonated chicken eggs for influenza virus propagation [50,52,53] and as a means of determining virus titers through plaque assays [49,54,55,56]. MDCK cells have also been used in almost 1000 papers to produce influenza vaccines [57,58,59], study virus pathogenesis and replication kinetics and evaluate the efficacy of anti-viral drugs [60,61]. A recent example for the usefulness of MDCK cells is the study of compound T-705 (now called favipiravir [62]). This compound was able to inhibit plaque formation in MDCK cells even up to 72 hours post infection (hpi) with A/Puerto Rico/8/1934 (H1N1) (PR8) virus. T-705 also inhibited viral growth at MOIs of 0.0001 to 1 and its activity was not affected by virus multiplicity. In cells that were treated with T-705 at 1 μg/mL, both supernatant and cells had up to 1000 times lower virus yields than non-treated controls. When the dose of T-705 was increased to 10 μg/mL, no productive infection was observed. The drug was then further tested in mice and is now approved in Japan for treatment of influenza infection [62].

#### 2.2.2. Immortalized Lung Cells, e.g., A549 and Calu-3

The A549 human lung adenocarcinoma epithelial cell line and the Calu-3, a human bronchial epithelial cell line, have been widely used to study influenza virus–host interactions [63,64,65,66,67,68,69,70,71,72,73,74,75,76,77,78], viral protein biochemistry [79,80], drug studies [81,82,83] and for performing large-scale screens, such as a CRISPR activation screen, to identify genes that inhibit influenza virus infection when they are overexpressed [82]. 

#### 2.2.3. HEK293 and Vero Cells

HEK293 cells are epithelial cells derived from embryonic kidney and were exposed to sheared fragments of adenovirus DNA. These cells are widely used for protein expression studies due to the ease of transfection into these cells [84]. Vero cells are kidney cells derived from *Cercopithecus aethiops* (African green monkey) kidney and have been extensively used in virology research [85,86]. Both HEK293 and Vero cells are being studied for basic research regarding influenza as well as for vaccine research (reviewed in Milian E., 2015 [44]).

### 2.3. Animal Models 

The use of animal models in influenza research has been extensively discussed in previous reviews [29,30,33,87,88,89]. In brief, mice [30], ferrets [35], guinea pigs, swine and non-human primates are commonly used to study influenza.

#### 2.3.1. Mice

The mouse is widely used because of its low cost, small size and ease of husbandry [30,33,90]. Mouse-specific tools for molecular biology and immunology studies are also readily available. Many inbred laboratory mice are also susceptible to influenza virus infection [30]. However, a significant drawback of the mouse model is that wild mice are in fact not susceptible to influenza because they express the antiviral protein Mx1 which is part of a GTPase family with known antiviral functions [91,92,93], and laboratory viral strains require prior adaptation to mice. As such, many laboratory mice strains do not display disease following infection with primary human influenza virus isolates [30]. This is, in part, due to the preference of human influenza A virus for α2,6-linked sialic acid receptors on the surface of the host cell. While humans express the α2,6-linked sialic acid receptor in the upper respiratory tract, where influenza virus commonly replicates, mice express the α2,3-linked sialic acid in both the upper and lower respiratory tract [89]. Moreover, mice do not display the same clinical signs during acute viral infection as they do not exhibit fever, unlike in humans [30]. 

#### 2.3.2. Ferrets

Ferrets rank among the best animal models of influenza. The lung physiology of the ferret is highly similar to that of humans [35]. They share many of the clinical symptoms presented by humans following influenza infection and are susceptible to the human influenza virus [88]. Thus, no prior adaptation of the virus to the ferret is necessary. Furthermore, the virus transmits easily among ferrets [88], recapitulating virus spread in humans. Given the similarity of the physiological response to influenza virus between ferrets and humans, ferrets have been used to study influenza proteins [94,95] and to assess the effectiveness of anti-viral compounds at ameliorating disease progression [89,96,97,98]. However, the limitation of ferrets is that while they are highly susceptible to infection, intramuscular or other immunization routes with noninfectious antigen results in the poor antibody response. In the light of the importance of immunogenicity studies of existing or novel vaccines, this is a huge drawback for the use of ferrets [87].

#### 2.3.3. Non-Human Primates (NHP)

Due to the similar genetics and physiology of monkeys to humans, non-human primates (specifically macaques) have similar immune responses to humans in regard to influenza infection ([99] and reviewed in Bouvier et al., 2010 [30]). Non-human primates are used to study highly pathogenic virus strains where the host cytokine response is believed to be involved in disease pathology and to study the efficacy of drugs and vaccinations against such strains. However, high costs, complex husbandry requirements and ethical issues make such primates less accessible for routine influenza studies [30]. To date, there has not been any transmission studies performed with NHPs. In addition, there are differences in virus binding sites in macaques and in humans for avian influenza [100]. 

#### 2.3.4. Pigs 

Since influenza A is the main cause of respiratory disease in pigs, this animal can be used as a model to study influenza (reviewed by Rajao et al., 2015 [31]). This is due to their similarities to humans in terms of genetics, anatomy, physiology and social behavior.Swine respiratory tract has similar tracheobronchial structure, lung physiology and size to humans [101,102]. The immune system of pigs is also similar to that of humans, but is not fully understood yet compared to that of other animal models. Limitations of such a model are the cost of husbandry and a larger space needed to house the animals compared to mice.

## 3. Novel, Alternative Models for Studying Influenza 

As mentioned above, mammalian models, animals and cell lines, are obviously biologically relevant for influenza studies but do possess serious limitations. Prominent drawbacks of these systems are their weak permissiveness for biochemical and genetic methods. In addition, the mammalian systems are intricate, putting significant obstacles in addressing the specific direct effects of the virus on cellular metabolome, proteome and transcriptome. Finally, these systems are not very handy for high-throughput screening for mutants, inhibitors and interacting proteins. 

Simple model organisms, such as yeast, flies and fish, are more open to these approaches and many ‘influenza questions’ have already been asked in them. Clearly, as the question of relevance to the human disease is a concern in these models, any finding must be further tested.

Below we provide a summary of how yeast, *Drosophila* and zebrafish have been used so far better understanding of influenza. In addition, the use of primary human cells derived from patients provides advantages and benefits that current immortal cell lines do not. As these cells are prepared from patients, they can be used immediately at the translational level. The primary cells are the only system that enables the study of patient factors, prediction of drug effects on individual patients (personalized medicine) and understanding of environmental and social factors on the disease. While many simple model organisms, such as *C. elegans*, have been used for other biological research, no influenza studies have been attempted in these models and will not be discussed in the review. 

### 3.1. The Yeast Saccharomyces Cerevisiae: A Powerful System for Identifying Cellular Targets of Viral Proteins, for Reconstituting Viral Machineries, for Drug Screening and for Antibody Production

The yeast *Saccharomyces cerevisiae* (*S. cerevisiae*) and *Scizosacchromyces pombe (S. pombe)* have been model organisms for studying all the basic cellular processes in the eukaryotic cell, including transcription and transcription regulation, translation, RNA processing, signal transduction and de novo (M12)(M13) synthesis of amino acids and nucleotides [103,104,105]. Important processes, such as cell cycle, autophagy, regulation of chemical energy (ATP), basic signaling pathways (e.g., MAP kinase cascades) and vesicle trafficking, were first discovered in yeast [106]. Most findings in yeast turned out to be relevant to all eukaryotic cells, including mammals, making *S. cerevisiae,* and *S. pombe* relevant and legitimate systems for studying all the basic biochemical, physiological, metabolic and genetic processes of the eukaryotic cell. *S. cerevisiae* is more widely used than *S. pombe* has been termed the ‘*E.coli* of the eukaryotic cell’ [107,108,109,110]. As the yeast biochemical machinery is so similar to that of mammals, in many cases, mammalian proteins can functionally replace the relevant yeast ortholog [111]. These observations made yeast an in vivo test tube in which to study foreign proteins such as the bacterial pathogenic factors [112,113], mammalian transcription factors and steroid receptors [114]. *S. cerevisiae* cells have also been used to reconstitute mammalian enzymatic complexes and biochemical pathways such as Ras and its regulators, MAP kinases, GCN2/PKR and even the olfactory pathway from the taste receptor to the activated enzyme [107,115,116,117,118,119]. As yeast has become a central system in biological studies, various sophisticated experimental tools have been developed. Some of them are not available yet in any other model organism, For example, one-step knockout and knock-in processes [120,121], the availability of a knockout library of all non-essential genes, the ability to harbor a foreign gene at the number of copies desired and the availability of libraries with all yeast genes tagged with various tags, including GFP. Furthermore, as *S. cerevisiae* is a simple single-cell organism with just about 6400 genes, advanced bioinformatics and system-biology tools are most powerful and accurate in yeast [111]. Importantly, *S. cerevisiae* have been used to study viral-induced apoptosis, viral mediated cell cycle regulation [10] and for drug screening [122].

Yet another type of yeast, *Pichia pastoris (P. pastoris)*, is also very useful for research, mainly for efficient expression of proteins [123]. It was used, as such, for studying influenza proteins, including the vRNP complex that was reconstituted in it. The three components of the viral polymerase complex (PA, PB1 and PB2) were expressed in *P. pastoris* by inserting all three genes into the chromosome [124]. The yeast cells were able to express the protein and when the viral polymerase was purified, the complex was able to synthesize RNA in vitro. Similarly, A/swan/Poland/305-135V08/2006 (H5N1) HA gene was expressed in *P. pastoris* and the protein was purified for mouse immunization. This recombinant HA was found to be effective in eliciting a high immune response in mice [125]. As *P. pastoris* is used mainly for large-scale protein production and antibody screening, it will not be further discussed here. 

The yeast model and the powerful tools that come with it have been assisting influenza research in several ways. A common approach is expressing the viral proteins in yeast and thus, studying the properties of single viral proteins in an isolated in vivo environment. In 1985, A/WSN/1933 HA was expressed in *S. cerevisiae*, and it was found that the HA protein was glycosylated and membrane bound [126]. Also, yeast cells expressing HA were found to lose the expressing plasmid overtime, a phenomenon that may indicate the protein’s toxicity. The yeast system was used to study structure–function relationships within the HA protein by expressing different deletion mutants. HA500 (lacking the C-terminal transmembrane anchor and cytoplasmic domain) and HA325 (containing only HA1 region and lacking the entire HA2 region) were glycosylated and stable compared to the other mutants [127]. However, HA500 was retained intracellularly, while HA325 was found to be secreted to the extracellular medium and was hyperglycosylated. This demonstrated the usefulness of yeast for studying structure-function relationships of important influenza proteins and the properties of mutated viral proteins. NS1 and NS2 were expressed via a copper inducible promoter [128,129] and NS1 expression was found to be toxic and to be localized to the nuclear fraction [128], while NS2 was similarly localized to the nucleus in yeast, but was not toxic [129]. Hence, yeast can also be used to study subcellular localization of the viral protein and its impact on cellular function. Expression of the M2 ion channel in yeast affected growth [130] and the use of a reversible M2 channel inhibitor, BL-1743, caused an increase in transmembrane proton flux due to the re-establishment of the proton gradient by M2. Interestingly, when the influenza PA-X protein was expressed in yeast, 29 unintended mutations appeared in PA-X. 24 of the PA-X mutants seem relevant to mammalian systems as they caused a reduced antiviral shutoff activity when tested in mammalian cells [131]. These models proved useful for characterizing the localization and function of the viral protein, for identifying potential mutation sites and for recognizing host processes affected by the viral protein. It seems, however, that the full potential of yeast as a model for understanding each viral protein has not been harnessed yet.

In cases where viral proteins impose an easily measurable phenotype on the yeast cells or in cases where the purified protein is an active enzyme, the systems could be used for the isolation of new inhibitors (molecules that reverse the phenotype or the activity) via high-throughput, rapid and efficient screens. Part of the Fujian H5N1 strain virus NA (HNA) was expressed in yeast and was shown to be functional and could be inhibited upon addition of oseltamivir and zanamivr [132], suggesting that the yeast-based system is functional for future screening of novel NA inhibitors. The toxicity imposed on yeast by of NS1 expression has indeed been harnessed for drug screening. Approximately 2000 compounds from the National Cancer Institute Diversity Set library were screened for compounds that would restore normal growth. Of the nine compounds identified, two were found to be toxic to MDCK cells and three showed no activity against influenza virus replication. Of the other four, three were able to activate anti-viral IFN-β in MDCK cells in a similar way to cells infected with delNS1 virus (virus lacking NS1) [133]. Equivalent screens took advantage of the growth inhibition phenotype induced in yeast by expression of M2 and compared the efficacy of the novel compounds to amantadine which led to new lead compounds against M2 [134,135]. Using the M2 yeast expression, 18 novel compounds were identified [135], while another high-throughput screen identified a spirothizamenthane compound, which was used to synthesize derivatives thereof. One of the derivatives, termed compound 63, was found to inhibit the native M2, and, importantly, also S31N- and V27A-mutated M2 protein, which are amantadine-resistant mutants [136]. The M2 yeast system was used together with MDCK cells to test the efficacy and toxicity of synthesized arylsulfonamide compounds so as to elucidate the structure–activity relationship [137]. 

The above studies demonstrate the efficiency of yeast for high-throughput drug screening in living cells, increasing the number of potential drugs in the pipeline. The same yeast systems could be used to study already known inhibitors and to identify mutations in the viral proteins that cause drug resistance (see in Hahnenberger et al., [130] and Kurtz et al., [134]). 

Another important use of the yeast model is to study human proteins known to be targets of the influenza virus. Many of these proteins have homologs in yeast, providing a convenient system for addressing their function in the simple eukaryote and deduce their role in the infection process in man. An example would be the Mx proteins. *S. cerevisiae* has a Mx-related GTPase known as Vps1, and another protein, Mgm1, both of which have a high similarity to Mx [138]. Mutational analysis of Vps1 in yeast has revealed key aspects of the protein structure-function [139]. Similarly, the function of Mgm1 was revealed in yeast through deletion studies [140]. 

Yeast could also be used to establish the entire influenza replicon to study, in an isolated system, the transcription and replication of the viral genome, polyadenylation [141], translational control [142], and post-Golgi vesicles, which are required for viral budding out of the cell [143]. 

As the effect of influenza on host machinery is so dramatic and involves interactions with a plethora of cellular components, one of the most challenging goals in the study of the virus is to identify the direct targets of each viral protein. By understanding which host processes are affected by each protein, specific inhibitors that target the viral-host interaction could be developed. This approach reduces the likelihood of resistance since host genes are unlikely to mutate as fast as viral ones. Many scientists have used the yeast two-hybrid system to identify host interactors of individual viral proteins: NS1 [144,145,146,147,148,149,150,151,152,153,154,155,156,157,158], NS2 [129,144,159,160,161], NP [162,163,164,165,166,167,168], PA [169,170,171,172], PB1 [173], PB2 [174], NA [175], M1 [176,177,178,179], M2 [180,181,182,183,184] and PB1-F2 [185,186,187,188,189]. Several studies applied the two-hybrid system to look at the vRNP complex [190,191] and the main eight viral proteins individually [192]. 

Given the occurrence of cell culture-selected mutations [193], using yeast as the alternative for determining the drug susceptibility of viruses may prove useful given that the proteins can be expressed intact with less likelihood of mutations. Given the benefits of yeast biotechnology, yeast can be used to identify and produce anti-influenza antibodies for vaccines and therapy [194]. Since yeasts have already been used to generate novel influenza vaccines [195,196], there seems to be untapped potential in using yeast to better elucidate the mysteries of influenza. 

### 3.2. Drosophila Melanogaster

#### 3.2.1. A Multicellular Organism which Serves As a Model for Development and Differentiation Studies and Is Useful to Study Viral Protein Functions and Host Immune Response

Unlike yeast, the fruit fly *Drosophila melanogaster* (*Drosophila*) is a multicellular organism. It has served, therefore, as a model for not only studying intracellular processes, but also for addressing the mechanisms of development and differentiation, and the functions of tissues and organs [197]. At the same time, similar to the yeast model, *Drosophila* is highly permissive to genetic manipulations, convenient and cheap to handle in the laboratory and has a short life span. These characteristics made it a pivotal model in biology and a variety of powerful experimental tools have been developed, including a library of mutants, mechanisms for tissue-specific expression, protocols for high-throughput screening for mutants and drugs, as well as the ability to control gene expression via RNAi [197,198,199]. Also, some *Drosophila*-derived cell lines are available, allowing scientists to address biological questions at the tissue-culture level in parallel to verification and advancing at the whole-organism level [200]. Taking advantage of the unusual properties of this fly, in combination with the effective experimental tools, it was possible to establish fly models for most critical human diseases. For example, expression in the fly of mutated α-synuclein caused loss of dopaminergic neurons and other effects that recapitulate the essential features of Parkinson’s disease [201] (reviewed in Aryal et al., 2019 [202]). Furthermore, genes that are defective in Alzheimer’s disease (e.g., Amyloid β; Tau) are conserved in *Drosophila,* allowing their study in the simple system [203,204]. The conservation of most processes responsible for metabolism and homeostasis, growth and development between *Drosophila* and mammals made it an excellent model for studying obesity, diabetes and aging (reviewed in Galikova et al., 2018 [205]; Inoue et al., 2018 [206]) and for investigating oncogenic pathways and cancer (reviewed in Mirzoyan et al., 2019 [207]; Enomoto, 2018 [208]). Notably, the concept of ‘tumor suppressors’ and the discovery of the first ‘recessive oncogene’ were established in *Drosophila* [209]. 

#### 3.2.2. Application of the *Drosophila* System for the Study of Viruses

The use of the *Drosophila* system for the study of viruses is not extensive yet, but is becoming more attractive (reviewed in Hughes et al., 2012 [11]). A common approach has been the expression of a viral protein in a particular organ of the fly and following the physiological, phenotypic or pathological effects. The expression of proteins of the Epstein-Barr and SARS viruses in the *Drosophila* eye, or of HIV proteins in the wings or in fat bodies led to the discovery of mechanistic aspects of the proteins and their partners within the host [210,211,212,213]. Many studies in *Drosophila* cells in culture have revealed the mechanism of action of viral proteins. This approach was useful, for example, for proteins of Hepatitis B, HIV and West Nile viruses [214,215,216]. Cells in cultures also served for large-scale screens, primarily for the identification of host genes required for viral replication and pathogenicity [217,218]. Some viruses were found capable of replication within the fly cells in culture or even within the whole fly (e.g., Dengue, Sindbis virus) [219]. MiRNA- and siRNA-mediated silencing of viral RNAs and antiviral-induced autophagy were major findings obtained through studying viruses in the fly [216,220,221,222,223,224,225]. 

#### 3.2.3. *Drosophila* and Influenza Virus Infection

Several of the influenza proteins have been studied in fly culture cells or in the whole fly. The influenza M2 was expressed in the wing and the eye, causing wing defects and a total loss of eye tissue. Males were found to have a more severe phenotype than females. Importantly, amantadine was shown to rescue the M2 phenotype, suggesting *Drosophila* as a reliable system for anti-influenza drug studies. As the expressed M2 was functional as an ion channel, transgenic flies were used to screen for mutations in ion transport or pH homeostasis-related genes of the host. Using flies with the relevant mutations and crossing them with the M2 transgenic flies, it was found that *vha44*, *vha55*, *vha68-1* and *vha68-2* were the strongest modifiers of M2-mediated phenotype. These four genes encode for the different subunits of the V_1_V_0_ ATPase. To confirm that the results obtained are not specific to flies, MDCK cells were treated with bafilomycin, a drug that targets the V_1_V_0_ ATPase, prior to influenza infection and this reduced infectivity [226]. Thus, this shows that the transgenic fly model is useful for studying M2 activity and allows the identification of important host targets of influenza M2.

The *Drosophila* model was used to report that influenza NS1 was able to suppress RNA-silencing-based antiviral response (RSAR), which is a host defense against RNA viruses [227]. A cell-based screen was performed using *Drosophila* cells to identify new RNA silencing suppressors. The coding sequence of GFP was used in-frame with the start of B2 ORF of flock house virus (FHV) RNA1 to obtain pFR1gfp. This was based on the previous observation that FHV B2 could inhibit the RNA interference (RNAi) pathway in FHV infection [228]. Using this technique, where GFP would be expressed once the RNAi pathway was silenced, it was observed that co-transfection of NS1 (A/WSN/1933 (H1N1)) plasmid with pFR1gfp plasmid could rescue GFP expression. This result shows that influenza NS1 functions as a RNA silencing suppressor through its N-terminal dsRNA-binding domain. It was shown that a single D92E mutation in NS1 increased the rescue efficiency of pFR1-ΔB2 (FR1 gene mutant that carried an untranslatable B2, similar to the pFR1grp) compared to WT A/NS1, which is associated with lethal H5N1 influenza A virus [229]. Using the same viral RNA silencing principle, five different RNA silencing inhibitors (including NS1A) were expressed using the GAL4 inducible expression system [199]. Upon induction of these transgenes in tissue-specific drivers, it was observed that NS1A was not an effective inhibitor of RNA silencing in transgenic flies. A later study reported that NS1 does not suppress RNAi in mammalian cells, an observation that supported the findings in *Drosophila* [230]. Using the same fly system to study NS1 function, NS1 cDNA from A/Vietnam/1203/2004 (H5N1) strain was expressed specifically in the wing. NS1 expression caused an increase of the distance between wing veins, a phenotype indicative of spatially broadened hedgehog (Hh) signaling. Several known mutations inserted into NS1 did not alter the Hh-modulating activity of NS1 in the fly’s wing. Yet, an unbiased mutagenesis screen provided a new NS1 mutation, A122V, located in the effector domain, which reduced the NS1-induced wing phenotype. Different Hh modulation among various viral strains exists (Udorn, swine flu and PR8), with the PR8 strain having the highest Hh modulation activity. Confirming the new effects of NS1, it was observed that Hh gene expression is altered in influenza-infected mice. In addition, the PR8-A122V virus was found to be more pathogenic than the PR8-WT virus in mice, suggesting that lower levels of Hh target gene induction may be protective against influenza infection [231]. 

An RNAi screen in *Drosophila* cell culture was used to identify new host genes required for viral replication and demonstrate the feasibility of using this system for genome screens in influenza [232]. Notably, a particular disadvantage of fly cells with respect to influenza is the fact that they do not express the influenza virus receptor α2,6-linked sialic acid, which is required for viral attachment to cells. Therefore, a modified virus, Flu-VSV-G, was designed, in which the HA and NA genes were replaced to encode the vesicular stomatitis virus (VSV) glycoprotein G. With these modifications, the influenza virus was able to enter *Drosophila* cells. A screen for host genes that affected virus-directed luciferase expression was carried out with Flu-VSV-G-R.luc (which expressed the *Renilla* luciferase gene) and an RNAi library against 13,701 *Drosophila* genes (~90% of all genes). A total of 176 of the screened genes inhibited luciferase expression, as identified in two independent tests. Several human homologues of encoding components in host machineries that are known to be involved in life cycle of the virus, e.g., *ATP6V0D1* (endocytosis pathway), *COX6A1* (mitochondrial pathway) and *NXF1/TAP* (mRNA nuclear export machinery) were selected by the authors showing the importance of the *Drosophila* system in the discovery of novel influenza virus–host targets. 

It seems that the use of *Drosophila* as a model and as an experimental tool for influenza studies is only in its infancy. However, the advances made so far using the fly system will undoubtedly contribute to the realization that the system is a relevant model for understanding important aspects of viral infection, and will lead to many more studies in the future. 

### 3.3. Zebrafish

The zebrafish *(Danio rerio)* belongs to the *Cyprinidae* family, the same family as the carp and the goldfish. It has been a model of choice that bridge the gap between lower animals and mammals. As vertebrates, zebrafish are closer to humans than fruit flies on the evolutionary scale. Compared to mice, they are easier to work with and study. The benefits of the zebrafish is that it has a fully developed immune system [233]. It also enables genetic screens [234] and real-time visualization, as seen with *M. marinum*, a bacteria species used to model tuberculosis (which is the same genus as *M. marinum*) in zebrafish [235].

Given that it has both innate and adaptive immunity, zebrafish can be used to study specific aspects of immunity development. During the initial 4 days of embryonic development, it exhibits no adaptive immunity markers and full functionality takes 4–6 weeks to develop [236]. Hence, it is possible to study the innate immune responses exclusively in the early developmental weeks [237]. Host-virus interaction studies have been performed in adult zebrafish for other viral infections such as chikungunya [238], herpes simplex virus 1 [239] and hepatitis C [240,241]. 

In the context of the influenza A virus (IAV), zebrafish embryos were reported to have α2,6-linked sialic acids, which enables the virus to enter and infect the embryos. Local infection of 5 days post-fertilization (dpf) embryos in the swimbladder resulted in zebrafish mortality. Moreover, antiviral genes were found to be induced in the fish, particularly *ifnψ1* (zebrafish virus-induced interferon) and *mxa* (an ortholog of the mammalian Mx protein [242]), following infection with IAV (PR8 and A/X-31 (H3N2)). Microscopy and histopathological analysis of the zebrafish found edema and tissue destruction similar to those observed in human infection. At 48 hours of infection with PR8, necrosis of the liver and gill tissues were also observed. With X-31 infection, there was necrosis of the renal hemopoietic tissue. Moreover, treatment with zanamivir, beginning with 3 hpi, was found to be protective against IAV infection by reducing mortality, viral replication and burden using a GFP-tagged NS1 in PR8. Hence, zebrafish can serve as a model for studying host-virus interaction and for drug screening [243].

2 dpf zebrafish were infected with PR8 and muscle fiber damage was observed [244]. Muscle damage seemed to worsen at 48 hpi compared to 24 hpi. A NS1-GFP virus showed that the virus infected skeletal muscles of the fish. Using transgenic *(fli1:gfp)* zebrafish, in which GFP expression was controlled by an endothelial-specific promoter, IAV increases vascular permeability and compromised sarcolemma integrity. There was also failure of the muscle fiber-ECM adhesion, which resulted in muscle fiber death. This effect could be due to pro-inflammatory innate immune signaling cascades being induced via the NFkB pathway (suggested by using NFkB reporter line *(NFkB:EGFP)^nc^*). Using a transgenic fish, *Tg(BACmpo:gfp)^i114^* where GFP expression was controlled by the neutrophil-specific myeloperoxidase (*mpo*) promoter, it was reported that neutrophils infiltrated muscle tissue and was localized to the unanchored end of retracted fibers after infection [244]. This could be mediated by the p38 MAPK, Rho/ROCK and PKC pathways [244]. Finally, using a zebrafish muscular dystrophy model *(sapje/dmd)*, infected zebrafish were found to have severe muscle degeneration in almost every muscle segment, with increased mortality at 4 and 5 dpi compared to the PBS injected mutant zebrafish. These studies clearly display zebrafish as a relevant model for influenza studies. 

### 3.4. Primary Human Tissue Culture

As described above, the main cell lines that have been traditionally used for studying influenza included the MDCK cells and cancer-derived lung cell lines such as A549 and Calu-3. HEK293 and Vero have also been used quite widely. The use of defined immortal cell lines that are readily infectable and in which influenza replicates is of obvious convenience. These systems contributed indeed significantly to our current understanding of influenza (reviewed in Powell et al., 2018,2018 [12,245]). Nevertheless, established cell lines possess major disadvantages. In addition to the inherent disadvantage of cells in culture that are disconnected from the organism’s systems (immune, endocrine, neuronal), established cell lines, particularly those that are oncogenically transformed, develop unique biochemical wirings and create their own repertoire of gene expression. Many cell lines are therefore significantly different from the original parental cells that were taken from the tissue and used to establish the cell line [246]. Such cells may respond to viral infection in a different manner than cells in the intact tissue. Primary cell cultures, on the other hand, provide both the advantage of the convenience of studies in culture and the advantage of cells that have not re-wired their biochemical networks and have not yet shed their identity. An unequivocal advantage of the primary culture approach is the fact that they could be prepared from patients. These patient-derived cells may come with known patients’ characteristics such as gender, co-morbidities, or lifestyle habits like smoking, allowing a personalized investigation of the disease. Amongst all the experimental models, the primary cells are in fact the only one that could be derived from patients and could provide the closest physiology to an actual human clinical subject. 

The ability of the primary culture to faithfully preserve properties of the original tissue is exemplified in the primary lung cells that secrete a class of proteases that can cleave influenza HA for viral entry [247], and when cultured at air-liquid interface (ALI), undergo polarization and produce mucus [248]. At ALI, the mucus readily forms about a week after air exposure and can shield the virus from escaping apically into the liquid medium. This forces the virus to travel laterally to the neighboring cell, similarly to the events occurred in a natural infection [12]. 

The use of primary culture allows close inspection of the very specific cell types that were preferentially infected by the virus. Different influenza viruses (human and avian source) tended to infect different human respiratory cells (nasal and trachea-bronchial cells) [249]. Human viruses infect non-ciliated cells while avian ones infect ciliated cells (tracheo-bronchial epithelial cells). This was attributed to different distribution of α2,6- and α2,3-linked sialic acids. For the 2009 pandemic H1N1 strain (H1N1pdm), low levels of viral replication was observed in Alveolar Type I cells (ATI) compared to normal human bronchial epithelial (NHBE) and nasopharyngeal epithelial (NPE) cells [250]. Both ATI cells and NHBE cells represent the lower respiratory tract, which is the site of potential influenza-related complications, while the NPE cells represent the upper respiratory tract that is involved in viral transmission. This demonstrates that primary cells can be used to study novel viral strains for virulence potential and transmissibility. Various respiratory tissues obtained from patients and human alveolar epithelial cells (AECs) have been used to understand the cellular tropism of different avian H5N6 viruses. Using ex vivo lung and bronchus cultures, all the H5N6 viruses in the study were able to infect these tissues. The viruses were able to infect both ciliated and non-ciliated cells in bronchial tissue and type-II AECs [251]. This may reflect the pathogenicity of H5N6 viruses to cause severe influenza in humans. 

#### 3.4.1. Human Nasal Epithelial Cells (NECs)

The first line of mechanical and immunological defense against pathogens in the upper respiratory tract is the nasal epithelium [252]. A holistic understanding of viral replication dynamics and immune responses generated in airway epithelial cells has not been completed yet [253]. Also, the ciliated cells of the proximal airways (such as the nose and trachea) are important for the spread and infection of the influenza virus [254]. 

Studies with primary human nasal epithelial cells (NECs) have significantly assisted our understanding of influenza in this tissue. For example, in normal tissue infected with influenza, eotaxin (eosinophil-specific chemoattractant) was found to be increased and released into the supernatant [255]. Moreover, these cells were found to generate intracellular reactive oxygen species (ROS) and had increased expression of nicotinamide adenine dinucleotide phosphate oxidases (Nox) [256]. One particular Nox that was found to be significant was Doux2 (Dual oxidase 2). 

Given that NECs can be derived from patients, certain social factors, such as smoking, can be studied specifically in such cells in relation to influenza. NECs derived from smokers expressed lower levels of anti-viral cytokine IFN-α and increased pro-inflammatory cytokine IP-6, resulting in increased influenza-induced cytotoxicity compared to NECs from non-smokers. [257]. In addition, smoking appeared to reduce cytokine IP-10 expression and release from NECs, while increasing Th2 chemokine thymic stromal lymphopoietin (TSLP) levels [258]. This shows that smoking could have an effect on the interaction between epithelial cells and immune cells. It should be noted that the use of primary cultures is the only way in which responses of smokers and patients to influenza infection could have been tested.

Adult human nasal epithelial stem/progenitor cells (hNESPCs) from nasal biopsy specimens of both healthy subjects and patients with allergic rhinitis have been established [259]. The in vitro- passaged hNESPCs retain the capability of full differentiation into human nasal epithelium cells (hNECs). When these hNECs were infected with the A/Aichi/2/1968 (H3N2) virus, the progeny virus could be detected from 16 hpi. Infected hNECs were found to elicit similar immune responses to other known models. Interestingly, there was individual variation among the patient-derived immune responses and apoptotic cell death, which is reflective of different responses to the same virus in a population. Infection of hNECs has uncovered novel host signaling mechanisms which the influenza virus can stimulate. For example, H3N2 infection of hNECs resulted in an increased expression of oncostatin M (OSM), which is part of the IL-6 type cytokine group [260] and upregulation of miRNAs such as miR-146a-5p, whose target genes include TRAF6 and IRAK1 [261]. Microarray analysis of infected hNECs has also revealed perturbations in homeostasis and metabolic pathways in response to influenza infection [262]. Other interesting observations using patient-derived NECs include the effects of estrogenic compounds on NECs derived from male and female donors, showing that estrogen exhibited protective effects upon infection in female, but not male, NECs. [263]. This reflects how hormones and gender may affect an individual’s response to influenza.

These studies combined, all performed on primary NECs, allow the establishment of the preliminary model describing the influenza-host relationships at the nasal epithelium. This new information is valuable as NECs are the first to come in contact with the virus and play a role in virus transmission. 

#### 3.4.2. Normal Human Bronchial Epithelial Cells (NHBE) 

Primary NHBE cells are derived from patient biopsies at the most distal aspect of the trachea and carina. While normally an upper respiratory infection, influenza is known to cause complications such as bronchitis in patients with chronic conditions, the elderly and children [21]. Hence, NHBE cells may provide further knowledge to influenza tropism, the host susceptibility to complications and response to the virus in the lower respiratory tract. 

To validate these cells as a model for influenza, NHBE cells were compared with MDCK cells and NHBE cells were found to have better correlative data in terms of replication abilities of live attenuated viruses isolated from different strains [54]. This reflects that NHBE cells were able to better model virus replication and infectivity of various viral strains from human and avian sources in humans compared to MDCK cells. Histological and electron microscopy studies revealed that NHBE morphology was similar to that of carina and distal trachea. The conjugated NY312-AF594 virus (A/NY/312/01) was shown to have similar binding patterns in NHBE cells as to those observed with human respiratory tissues. The replication kinetics of pandemic 2009 virus strain (A/CA/04/2009 (H1N1)) were similar to the NY312 virus in NHBE [264]. 

Given that NHBE cells are appropriate to study viral cellular tropism, several studies have used them to compare various avian influenza strains [265,266,267,268]. NHBE cells predominantly display α2,6 linkage sialic acids receptors as well as α2,3 sialic acid receptors [269,270]. Since avian viruses generally bind to α2,3 receptors [271], using such cells would provide a better predictive ability of a novel viral strain in causing severe influenza in humans. 

To better understand the effects and functions of viral proteins in infection, reverse genetics-derived viruses carrying target mutations in NP and/or PB2 proteins have been used [272]. Reverse genetic performed with A/Duck/Alberta/35/1976 (H1N1) showed synergistic effects between NP and PB2 mutations. Infected NHBE cells were shown to have lower conductance of the chloride ion channel cystic fibrosis transmembrane regulator (CFTR) [273]. This may be due to the virus targeting the CFTR for early lysosomal degradation and affecting ion transport in the host. In the case of viral HA, various mutations affected the virus’ ability to infect cells. This was seen with the H9N2 virus [274], H5N1 virus [275] and the pandemic 1918 strain [276]. HA mutations were also studied in relation with neuraminidase inhibitor resistance [277]. Studies with NHBE cells also showed that decreased neuraminidase activity was required for H5N1 virus adaptation to the human airway epithelium [278]. Studying both HA and NA, viruses found to have avian-like surface glycoproteins exhibited reduced infectivity at 32 °C in NHBE, which would be required for human-to-human transmission [254]. 

NHBE cells have been used to provide a better understanding of the host response to influenza infection in the bronchial tissues. Proteomic studies performed with human NHBE infected in vitro have revealed signaling pathways which could be important, including RhoA-associated signaling pathway (RhoA is a GTPase that regulates actin cytoskeleton[279]) and reduced Hippo signaling (required to suppress virus-induced interferon production) [280]. Similarly, proteomic analysis on commercially obtained NHBE cells (Lonza) infected with PR8 [281] identified proteins which were associated with host cell defense response, endocytosis and GTPase activity. Influenza virus was also found to increase apoptosis and pyroptosis [282], as well as c-Myc, glycolysis and glutaminolysis in NHBE cells from pediatric patients [283]. Importantly, NHBE cells are also being used for immediate translation of culture tests to clinical uses. For example, TVB024 (a small molecule inhibitor against ATPase) was tested and found to inhibit viral replication in patient NHBE cells [284]. The results were confirmed in bronchial explants obtained from the same patients. Using TVB024 prophylactically, the combination of TVB024 and oseltamivir reduced viral shedding and this was more effective than oseltamivir alone in inhibiting epithelial infection. In addition, infected NHBE cells were used to predict patients’ response to H7N9 infection [285]. Viral RNA levels in cells taken from older patients (≥65 years) were significantly lower compared to young patients. Obese patients tended to have increased viral RNA levels but lower cytokine levels. Finally, NHBE cells were used as an ex vivo model to test DAS181 efficacy in removing sialic acid and inhibiting influenza infection [286]. Given these results, DAS181 has currently reached phase II clinical trials [24]. 

Thus, NHBE cells isolated from patients are a rich source of information in understanding what host factors affect influenza. They also point at the viral factors which increase the virus’ ability to infect and propagate in these cells, increasing the chance of viral bronchitis. Importantly, NHBE cells are an unequivocal tool for predicting the patient’s response to drugs and treatment and are therefore a most valuable tool for personalized treatment of influenza patients. 

#### 3.4.3. Human Alveolar Epithelial Cells (AECs)

The alveolar epithelium makes up the gas exchange portion of the lung, which is the main target for influenza A pneumonia. The epithelium is made up of two main cell types, known as type I and type II cells. Alveolar Type I cells (ATIs) are required for the gas-exchange surface in the alveoli [287]. Alveolar type II cells (ATIIs), also known as type II pneumocytes, synthesize and secrete pulmonary surfactant, secrete chemokines and cytokines, and are involved in the innate immunity of the lung [288]. The different cell tropism between various strains of influenza was described, but it was found that the viruses tested could only be found in type II but not in type I pneumocytes [289]. While A549 cells have been used to study ATII physiology, A549 cells mimic ATI cells more than ATII cells [290]. Primary AECs (either ATI or ATII) exhibit a normal cellular physiology and also carry individual characteristics from the patients they were derived from. 

Several studies have focused specifically on ATI cells, revealing an important transcription factor Nrf2 in the host-response to influenza, inducing the transcription of HO-1, important in the antioxidant defense system [291]. Overexpression of Nrf2 protected cells from infection as it decreased viral replication and increased IL-8 expression, which may be due to increased GSH levels observed with Nrf2 overexpression. To study viral effects of specific mutations, recombinant viruses of NY1682 with PB2-D701N were used to infect ATI cells [292]. The NY1682-D701N was observed to have higher titers than the wild type virus and there were lower levels of IFN-λ, which represents an important antiviral type III interferon in ATI cells. While the PB2-D701N substitution has been shown to cause increased viral replication in mice and guinea pigs [293,294,295], using ATI cells prove that this mutation also increases viral replication in human cells. 

Studies have also used ATII cells isolated from human lungs, and these cells were shown to support influenza infection with PR8 and A/Philippines/1982 (H3N2) [296]. ATII cells produced inflammatory cytokines such as IL-6, IL-8, MCP-1 and RANTES. Interestingly, IL-29 was found to be the main cytokine produced by infected ATII cells. 

AECs were shown to be primarily targeted by H1N1 viruses (A/California/07/2009, A/New York/1682/2009 (NY1682), A/California/04/2009 (CA04)) [297]. AECs from different donors had different susceptibilities to the viruses. Obese donors showed significantly increased infection rates compared to non-obese donors. The link between infection rates and obesity could have been monitored only in the patients-derived primary cultures. HA sequences were studied and the A214T substitution decreased NY1682 infectivity of AECs significantly which could explain the differences observed between the various H1N1 viruses. In the context of H5N1 and H1N1 infections [298], the A/Hong Kong/483/1997 (H5N1) (HK97) and A/Hong Kong/54/1998 (H1N1) (HK54) could infect and replicate in both ATI-like or ATII cells. Using specific cell populations and co-cultures with other cell types, different host processes can be elucidated in the context of influenza to further understand host–virus interactions in specific cell types. Examples of novel host processes in ATII cells are the activation of epidermal growth factor receptor (EGFR) [299], increased AIM2 signalling [300], increased expression of CEACAM1, a cell adhesion molecule [301], and the reduced secretion of surfactant proteins, SP-A and SP-D [302]. While some of these processes are known in influenza infection, these studies with AECs confirm the role of these processes in influenza. 

In summary, primary human tissue cultures open a new window into specific effects of the virus and, perhaps much more important, into patient-specific effect and patient-specific response to drugs and treatment. These systems are thus of great value not only for basic research, but also for immediate patient-specific translational therapeutic values.

## 4. Conclusions and Future Directions 

Although it may seem not relevant on a first glance, when studying aspects of influenza infection in yeast, *Drosophila* and zebrafish provide knowledge and a conceptual understanding that could not have been obtained in other experimental systems. The use of primary cultures, primarily with patient-derived cultures, is also of great value for personalized medicine and for understanding social and behavioral aspects of the disease. In spite of this understanding and in spite of the proven achievements, alternative models still remain underutilized. In fact, the *Drosophila* and zebrafish systems are in their infancy of contributing to the field of influenza research and the vast potential they hold in helping better understand of influenza awaits more extensive use of these models. Most of the studies carried out so far in yeast and *Drosophila* were specific to a given viral protein or for a specific host protein. And yet, only several of the influenza proteins have been expressed in yeast, flies and fish. In addition, the whole virus has yet to be reconstituted in yeast. 

Nevertheless, a few systematic large-scale experiments aimed at looking at the entire process of viral infection in model organisms have been performed [303], and we predict that they will lead the way to wider uses of the ‘alternative system’ that may ultimately lead to a deeper understanding of the virus and its pathogenicity and to a better treatment or vaccination. While the traditional models of influenza have their advantages and usefulness, we believe that the alternative models must be used in parallel. Table 1 shows the advantages of each alternative model. It is anticipated that many molecular mechanisms of influenza and new therapies against this long-standing malady will be revealed through their use. 

## Figures and Tables

**Table 1:** Comparison of the various novel methods of influenza.

	Yeast	Drosophila	Zebrafish	Primary Human Tissue Culture		
				NECs	NHBE	AEC
Advantages	Smaller genomes compared to higher eukaryotes Genome-wide single gene deletion yeast strain libraries and/or genomic DNA or cDNA plasmid libraries are available [10] Easily manipulated with various molecular, cellular processes [10] Easy to culture	Smaller genomes compared other eukaryotes Availability of genetic resources to study molecular mechanisms [11]	Closer to humans as they are a vertebrate model Has fully developed immune system [233] Enables real time visualization and genetic screens [234] Easy whole organism model to work with compared to mice	More physiologically relevant as these cells secrete proteases that can cleave influenza HA for viral entry [247] Cultured at ALI which mimics a natural infection by shielding a virus from escaping into the liquid medium [12] Able to enable analysis of patient characteristics that the cells are derived from		
Compared to known cell models of influenza	Yes	Yes	No	Yes	Yes	Yes
Compared to known animal models of influenza	No	Yes	No	No	No	Yes
